# Benzodiazepine use in Sao Paulo, Brazil

**DOI:** 10.6061/clinics/2020/e1610

**Published:** 2020-07-06

**Authors:** Angela Maria Campanha, Beatriz Ravagnani, Igor André Milhorança, Márcio Antonini Bernik, Maria Carmen Viana, Yuan-Pang Wang, Laura Helena Andrade

**Affiliations:** I Nucleo de Epidemiologia Psiquiatrica (LIM-23), Departamento e Instituto de Psiquiatria, Faculdade de Medicina FMUSP, Universidade de Sao Paulo, Sao Paulo, SP, BR; II Departamento de Farmacia, Universidade Estadual de Maringa, Maringa, PR, BR; III Instituto de Matematica e Estatistica, Universidade de Sao Paulo, Sao Paulo, SP, BR; IV Programa de Ansiedade, Departamento e Instituto de Psiquiatria, Faculdade de Medicina FMUSP, Universidade de Sao Paulo, Sao Paulo, SP, BR; V Departamento de Medicina Social, Programa de Pos-Graduacao em Saude Coletiva, Centro de Estudos e Pesquisa em Epidemiologia Psiquiatrica (CEPEP), Universidade Federal do Espirito Santo, Vitoria, ES, BR

**Keywords:** Psychiatry, Pharmacy, Psychotropic Drugs, Hypnotics and Sedatives, Benzodiazepines

## Abstract

**OBJECTIVES:**

To report the prevalence and factors associated with the use of benzodiazepines in the general population and those with a mental health condition in the metropolitan area of São Paulo, Brazil.

**METHODS:**

5,037 individuals from the Sao Paulo Megacity Mental Health Survey data were interviewed using the Composite International Diagnostic Interview, designed to generate DSM-IV diagnoses. Additionally, participants were asked if they had taken any medication in the previous 12 months for the treatment of any mental health condition.

**RESULTS:**

The prevalence of benzodiazepine use ranged from 3.6% in the general population to 7.8% among subjects with a mental health condition. Benzodiazepine use was more prevalent in subjects that had been diagnosed with a mood disorder as opposed to an anxiety disorder (14.7% *vs.* 8.1%, respectively). Subjects that had been diagnosed with a panic disorder (33.7%) or bipolar I/II (23.3%) reported the highest use. Individuals aged ≥50 years (11.1%), those with two or more disorders (11.2%), those with moderate or severe disorders (10%), and those that used psychiatric services (29.8%) also reported higher use.

**CONCLUSION:**

These findings give an overview of the use of benzodiazepines in the general population, which will be useful in the public health domain. Benzodiazepine use was higher in those with a mental health condition, with people that had a mood disorder being the most vulnerable. Furthermore, females and the elderly had high benzodiazepine use, so careful management in these groups is required.

## INTRODUCTION

Since its introduction in the early 60s (1), benzodiazepines (BZDs) have been the most prescribed psychotropic medication worldwide (2), despite their various therapeutic and side effects (2,3). Therapeutic indications for the use of BZDs are diverse and include the treatment of seizures (4), alcohol and barbiturate withdrawal symptoms (5), psychomotor agitation (6), insomnia and other sleep disorders (7), panic disorders (8), social phobia, generalized anxiety disorder (9), and as an adjunctive treatment for both depression and mania (10). Common side effects of BZD are drowsiness associated with incoordination or ataxia, which may lead to car accidents, problems with operating machinery, and, especially among the elderly, falls (11). Memory impairments that are potentially non-reversible have also been observed (3,12). Long-term use of BZDs is related to physical dependence. Discontinuation from chronic BZD use can result in withdrawal syndrome, particularly among the elderly (10). Withdrawal symptoms that have been reported include anxiety, sleep disturbance, irritability, a hand tremor, and rarely, more severe conditions such as seizures and psychosis (13).

Current guidelines such as the National Institute of Health and Care Excellence (NICE) (14) recommend that BZD should be used at the lowest possible dose for the shortest period possible. There are considerable evidence-based concerns regarding the serious adverse consequences of BZD use, such as falls (14), risk of suicide, abuse, dependence (10), and risk of Alzheimer's disease (15). In a series of pharmacoepidemiological studies conducted by the World Mental Health Survey Initiative (WMHS) (16,17), the use of psychotropic agents was evaluated in the general population. In addition, if a respondent had been diagnosed with a psychiatric disorder in the 12 months preceding the survey, this was recorded (18,19). The observed prevalence of BZD use in the general population ranged between 3.2% and 18.6% (Table 1). These rates were even higher among individuals that had been diagnosed with a psychiatric disorder, with a range between 9.2% and 41.9%. Generally, the prevalence of BZD use was higher among subjects with a mood disorder as opposed to an anxiety disorder. High consumption of BZD was also observed among females and older people (16,17,18,19,20). However, methodological diversity hampered a direct comparison of the rate of BZD use among participant countries of the WMHS Initiative.

Several studies on BZD use have been conducted in the non-developed regions. In Chile, the estimated prevalence of BZD use in the general population was 4% (21) (Table 1). Few studies have been conducted on the prevalence of BZD use in Brazil (20). The relationship between BZD use and mental health disorders in the general population has rarely been investigated (22). The reported prevalence of BZD use over one month in the general population was 2% and 3% in Rio de Janeiro and São Paulo, respectively (23). Among individuals who had been diagnosed with a mental health disorder, the one-month prevalence of BZD use was lower in Rio de Janeiro than in São Paulo (3.4% *vs*. 7.1%, respectively). However, the methodological differences regarding the period investigated, sample characteristics, and data collection preclude any direct comparisons being made (20). There is a lack of knowledge regarding the use of BZDs over a period longer than 12-months, its monotherapy or polypharmacy patterns, the prevalence of BZD use in specific mental health disorders, and the impact of BZD use on symptom severity, comorbidities, health insurance coverage, and health service use.

Given the scarcity of epidemiological data, we aimed to report the prevalence of BZD use in a representative sample of the general population and those with a mental health condition (diagnosed in the last 12 months) in São Paulo, Brazil. Information about monotherapy and the combined use of BZDs along with its relationship to symptom severity, comorbidities, health insurance coverage, and health service use are also discussed.

## METHODS

### São Paulo Megacity Mental Health Survey

Data for this report were sourced from the São Paulo Megacity Mental Health Survey (SPMHS). The SPMHS is the Brazilian segment of the World Mental Health Survey Initiative, coordinated by the World Health Organization and Harvard University. It was conducted in more than 28 research centers around the world. The SPMHS is a cross-sectional, population-based study. It was designed to estimate the prevalence of mental health disorders, mental health services, and psychotropic drug utilization in a representative sample of the general population. By design, individuals over 18 years old, living in the São Paulo metropolitan area were interviewed by trained lay interviewers (24).

### Sample

A sample of 5,037 individuals (response rate: 81.3%) were assessed using the Composite International Diagnostic Interview (CIDI), which generates DSM-IV diagnoses. We report on a subsample of 2,935 subjects who were submitted to a more extended version of the interview, which included questions on psychotropic drug use (24).

### Data collection

Participants were asked about prescription medicines that they had used in the previous 12 months for emotional issues, nerves, mental health, substance use, energy, concentration, sleep, or stress. According to the Anatomical Therapeutic Chemical (ATC) index 2018 (https://www.whocc.no/atc_ddd_index/), the medicines focused on in this report were anxiolytics (alprazolam, bromazepam, clobazam, chlordiazepoxide, cloxazolam, diazepam, and lorazepam), hypnotics and sedatives (chloral hydrate, flunitrazepam midazolam, zolpidem), and antiepileptics (clonazepam). The term “benzodiazepines” (BZDs) will be used henceforth to refer to all the above medicines.

### Data analysis

The data analysis examined both the prevalence of BZD use in the general population and among individuals who had been diagnosed with a mental health disorder. Diagnostic categories included in the analysis were anxiety, mood, substance use, and impulse-control disorders. Other clinical information included in the analysis was related to comorbidities and symptom severity.

Socio-demographic information collected included age, sex, education, family income, marital status, and employment status. Information about service use and health insurance was also analyzed.

The factors associated with BZD use were explored through a logistic regression analysis. The data analysis was performed using Statistical Analysis System (SAS).

## RESULTS

The prevalence of BZD use in the general population in the previous year was 3.6%. Diazepam (1.3%) and clonazepam (0.8%) were the most frequently used BZDs. Females used BZDs more often than males (5.5% *vs*. 1.6%). The use of BZD was also higher among subjects aged over 65, compared to those aged 50-64 and 18-24 years (7.8% *vs*. 6.1% *vs*. 1.8%, respectively) (Table 2).

The use of BZD monotherapy was reported in 1.8% of the sample. Antidepressants (1.4%) were the most commonly used psychiatric medication in combination with BZD (Table 3).

Table 4 presents the correlates of BZD use according to the socio-demographic variables, psychiatric diagnoses, comorbidities, symptom severity, use of health services, and the possession of private health insurance coverage.

The use of BZD was higher in those aged between 35-49 years (10.2% *vs*. 4.7%; OR=2.3; 95%CI=1.1-4.7), and over 50 years (11.1% *vs*. 4.7%; OR=2.6; 95%CI=1.2-5.3), than those between 18-34 years (4.7%). The use of BZDs was also higher among homemakers, retired subjects, and the unemployed compared to employed individuals (11.8% *vs*. 10.1% *vs*. 5.9%, respectively (Table 4).

Concerning psychiatric disorders, individuals diagnosed with a mood disorder (14.7%; OR=5.7; 95%CI=2.5-13), anxiety disorder (8.1%; OR=3.5; 95% CI=1.6-7.8), or substance use disorder (7.9%; OR=2.9; 95%CI=1.5-5.7) were more likely to use BZD than those without these disorders (Table 4).

Psychiatric comorbidities and symptom severity also play a role in the use of BZDs. Although individuals who had been diagnosed with two or more disorders used more BZDs than those with a single diagnosis (11.2% *vs*. 5.6%; OR=2.1, 95%CI=1.3-3.5), the likelihood of using BZD was lower in the adjusted model 2 (OR=0.4; 95%CI=0.2-0.9). The likelihood of BZD use was higher among patients with disorders that were considered to be serious or moderate than among those with a mild disorder (10.0% *vs*. 3.7%; OR = 2.8; 95%CI=1.7-4.8) (Table 4).

There was a trend (*p* = 0.0505) of higher BZD use among individuals who had health insurance coverage than those who did not (10.7% *vs*. 6.1%; OR=1.9; 95%CI=1.0-3.4). Remarkably, BZD use among individuals who reported using psychiatric services was almost 30 times higher than those who did not (29.8% *vs*. 1.3%; OR=25.0; 95%CI=13.7-45.6) (Table 4).

BZD use among subjects who had been diagnosed with a mental health disorder was 7.8%. Among the diagnostic classes, mood disorders displayed the highest prevalence of BZD use (14.7%). Participants who had been diagnosed with a panic disorder or bipolar disorder (33.7% and 23.3%, respectively) reported using BZD the most (Table 5).

The likelihood of BZD use was also higher among those with obsessive-compulsive disorder (OR=7.0; 95%CI=1.6-30.0), drug abuse (OR=8.2; 95%CI=1.9-36.4), drug dependence (OR=9.3, 95%CI=1.5-58.8), impulse control disorders (OR=5.6, 95%CI=1.1-27.7), and attention deficit disorder (OR=17.5, 95%CI=2.1-146.8) (Table 5).

Subjects that had not been diagnosed with a mental health disorder reported infrequent BZD use (1.9%). This prevalence was much higher among females than males (OR=13.0; 95%CI=4.1-41.3) (Table 5).

Considering the number of psychotropics used, 3% of subjects that had been diagnosed with a mental health disorder reported using BZD as a monotherapy. This was most frequent in those who had been diagnosed with attention deficit disorder (10.1%). The mean frequency of monotherapy was 2.9% for anxiety disorders, and 9.8% for panic disorders. Lower rates of BZD use were observed among individuals with mood (4%), bipolar I/II (4.5%) or major depressive disorders (4%) (Table 5).

## DISCUSSION

The 12-month prevalence of BZD use in the São Paulo metropolitan area was 3.6%. This rate is similar to that reported in a survey conducted in Rio de Janeiro (22). Similarly, in Chile, about 4% of individuals reported using hypnotics and anxiolytics (21). Conversely, the reported prevalence of BZD use in European countries (9.8% (25), 12.3% (16), 5.5% (19)), and the United States of America (5.2% (26)) is higher.

Even though the methodologies used were different, several studies have reported higher BZD use in Brazil previously. In 1979, the reported use of BZDs in São Paulo was 8.8% (27). Additionally, in 1993, 8.0% used tranquilizers and 1.2% used hypnotics (28). Recent studies have shown that the prevalence of use has indeed decreased to 1.6% and 2.7%, respectively, in Rio de Janeiro (22) and São Paulo (23).

The higher prevalence of BZD use among females may be due to females having a higher rate of mental health disorders, such as anxiety, major depression, and dysthymia (24). This sex difference persists even among individuals with a psychiatric diagnosis and among those without any psychiatric diagnosis. This suggests that other factors might be involved. Accordingly, the higher use of psychotropic drugs by females could also be explained by treatment-seeking behavior and lower alcohol and psychotropic drug use (25).

People working at home and those with low social functioning, such as retirees and the unemployed, also reported higher BZD use. This is in line with previous reports in Europe (29). In the current study, the use of psychiatric services increased the chance of using BZDs by 30%. Seeking help for emotional problems appears to be associated with the use of BZDs (29).

A surprising finding is the higher use of BZD among those subjects who had been diagnosed with a mood disorder compared to those with an anxiety disorder (14.7% *vs*. 8.1%, respectively), even regarding monotherapy (4.0% *vs*. 2.9%). However, this finding has been reported in a number of studies that have used a similar methodology (16). Sometimes, the use of BZDs among subjects with mood disorders has been comparable (16) or higher than the use of antidepressants (18,29). The non-specific effects of BZD appear to be less harmful than first-line antidepressants, which has prompted some clinicians to prefer BZD (10). In France, the use of hypnotics and anxiolytics was similar for those with depression or an anxiety disorder (43.4% *vs*. 42.5%). This finding reflects the challenges in diagnosing and managing mood disorders in primary care (17).

There was also increased use of BZDs in patients with more severe psychiatric disorders. One explanation for this could be the prescribing habits of clinicians. Usually, clinicians might include an adjunctive medication, such as BZD, for non-responders to treat residual symptoms such as insomnia and anxiety.

BZD is not considered to be the first-line treatment for most anxiety disorders, such as generalized anxiety disorder, phobias, and post-traumatic stress disorder, with antidepressants and antiepileptic drugs, usually prescribed (14). Nevertheless, the use of BZDs was also higher (18,25,29) or similar to the use of antidepressants among individuals with an anxiety disorder (16). It appears that in Brazil, patients are not receiving the most appropriate treatment option (22) because the use of BZD as a monotherapy was higher than that of other classes of psychotropic medications among subjects who had been diagnosed with an anxiety disorder. General practitioners issued 46.9% of the BZD prescriptions (28). Other specialists, such as cardiologists (15.3%) and neurologists (4.5%), issued more tranquilizer prescriptions than psychiatrists (11.7%).

The reported higher use of BZD in the elderly is in line with the patterns observed in most studies conducted in the United States of America (26), Canada (30), and Europe (25). In a systematic review (31) on inappropriate prescriptions for long-term BZD use and analogous non-BZD z-drugs, psychological dependence, absence of social support, ignorance about treatment options, withdrawal symptoms, and unfamiliarity with the potential side effects were the main drivers that perpetuate their use. Additionally, previous use was one of the main factors associated with the likelihood of BZD use among older patients (32). People from older cohorts that have been extensively exposed to BZD in their youth may become addicted (32), and become chronic users (32). Other factors included chronic illness, stress, pain, and insomnia (26). The higher BZD use in older cohorts is concerning due to older individuals being more at risk of falls (33,34,35), associated with healthcare utilization and decline in functional status (36). The causative effect of BZD on the risk of dementia is also a major concern (37). The literature suggests that long-term exposure to BZD is associated with an increased risk of Alzheimer’s disease (38,15), without stringent confirmation (39).

Taken together, our data and the existing literature must be urgently reviewed by governments, policymakers, and medical societies. There is some consensus that BZD should be discontinued in subjects aged 65 years or older. The most recommended deprescribing strategy for long-term BZD and Z-drug use is pharmacologic interventions. Multidisciplinary reduction of BZD and Z-drug exposure with the addition of alternative pharmacological therapies, psychological therapies (anxiety management, stress management, and psychotherapy), mixed programs (psychological therapy, gradual dose reduction, and usual care), and psychological education are some of the recommended approaches. These interventions present numerous, heterogeneous, and poorly described results, suggesting that studies are needed on how to best deprescribe BZD and Z-drugs in the future (40).

## CONCLUSION

According to the findings in the current study, the use of hypnotics and sedatives, which are mostly composed of BZD, has been declining over the last few decades in Brazil. Those that were older, female, or had lower social functioning tended to have higher BZD use. Subjects diagnosed with a mood disorder were more likely to use BZDs than those with an anxiety disorder. Individuals with disorders that were considered to be moderate or severe, those that used psychiatric services, and those with health insurance coverage tended to have higher BZD use. It is a public health challenge to find a surrogate for BZD and manage the existing chronic users.

## AUTHOR CONTRIBUTIONS

Campanha AM was responsible for the conception, draft, statistics, critical intellectual contribution. Ravagnani B was responsible for the draft and critical intellectual contribution. Milhorança IA was responsible for the statistics and critical intellectual contribution. Bernik MA was responsible for the conception, draft and critical intellectual contribution. Viana MC was responsible for the data acquisition and critical intellectual contribution. Wang YP was responsible for the draft and critical intellectual contribution. Andrade LA was responsible for the conception, data acquisition, statistics and critical intellectual contribution.

## Figures and Tables

**Table 1:** Pharmacoepidemiological studies conducted within the World Mental Health survey initiative and other studies in South America.

						Prevalence of use	
Reference	Location	Period	Names of studies	Sample	Age	General population	12-month diagnosis
Alonso et al. (25)	Europe	2001-2003	ESEMeD^a^	21,425	≥18	9.8%	25.5%
Bruffaertes et al. (16)	Belgium	2001-2002	ESEMeD^a^	2,419	≥18	12.3%	25.5%
Codony et al. (18)	Spain	2001-2002	ESEMeD^a^	5,473	≥18	11.4%	32.7%
Campanha et al. (20)	Brazil	2005-2007	SPMHS^b^	2,935	≥18	3.6%	7.8%
Gasquet et al. (17)	France	2001-2003	ESEMeD^a^	2,894	≥18	18.6%	41.9%
Grinshpoon et al. (19)	Israel	2003-2004	INHS^c^	4,859	≥21	3.2%	9.2%
Other studies in South America							
Rojas et al. (21)	Chile	1996-1998		3,870	16-64	04%	-
Quintana et al. (22)	Rio de Janeiro	2007-2008		1,208	≥15	1.6%	3.4%
Quintana et al. (23)	São Paulo	2007		2,536	15-75	2.7%	7.1%

**Table 2:** Prevalence of benzodiazepine use in the previous 12 months in the general population according to sex and age. São Paulo Megacity Mental Health Survey (N=2935).

Sex		N (%)	SE	*p*-value
Total		162 (3.6)	0.5	0.0004
	Female (N=1697)	122 (5.5)	0.9	
	Male (N=1238)	40 (1.6)	0.3	
Age				0.0687
	18-24 (N=406)	09 (1.8)	0.9	
	25-34 (N=684)	18 (2.6)	0.7	
	35-49 (N=1,026)	68 (3.4)	0.6	
	50-64 (N=590)	48 (6.1)	1.4	
	≥65 (N=229)	19 (7.8)	3.5	

Weighted proportions.

**Table 3:** Prevalence of monotherapy and combined use of benzodiazepines in the previous 12 months in the general population by sex. São Paulo Megacity Mental Health Survey (N=2935).

	N (%)	SE	OR (95% CI) female/male	*X^2^*	*p*-value
Benzodiazepines^a^	162 (3.6)	0.5	3.7 (2.0-6.7)	18.2	<0.0001
Monotherapy^b^	65 (1.8)	0.4	3.6 (2.0-6.5)	18.5	<0.0001
Combined use^c^					
Benzodiazepines +antidepressant	79 (1.4)	0.2	6.2 (2.3-16.7)	13.3	0.0003
Benzodiazepines + antipsychotics	11 (0.2)	0.1	11.3 (2.7-48.0)	10.9	0.001
Benzodiazepines + mood stabilizer	20 (0.4)	0.1	1.5 (0.5-5.2)	0.5	0.4904

Weighted proportions. OR, odds ratio; CI, confidence interval. ^a^At least one psychotropic drug. ^b^Only benzodiazepines. ^c^Any benzodiazepine drug plus another psychiatric medication. Sex comparison: males were used as the reference group.

**Table 4:** Correlates of benzodiazepine use in the previous 12 months with sociodemographic variables, mental health disorders, disorder severity, comorbidities, use of health services, and the existence of private health insurance coverage. São Paulo Megacity Mental Health Survey (N=1,271).

			Model 1		Model 2		
Variable	Total sample	N (%)	OR (95% CI)	*p*-value	OR (95% CI)		*p*-value
Sex							
Female	836	85 (8.7)	1.5 (0.7-3.2)	0.3563			
Male	435	32 (6.2)	1				
Age (years)				0.0418			
18-34	481	20 (4.7)	1				
35-49	472	56 (10.2)	2.3 (1.1-4.7)	0.0197			
≥50	318	41 (11.1)	2.6 (1.2-5.3)	0.0127			
Education (years)				0.4799			
Low (0-4)	346	36 (9.5)	1.3 (0.7-2.4)	0.421			
Low-average (5-8)	330	28 (6.8)	0.9 (0.5-1.8)	0.7639			
High-average/high (≥9)	595	53 (7.5)	1				
Family income				0.3014			
Low (≤0,5)	344	18 (5.5)	0.5 (0.2-1.0)	0.0641			
Low-average (0.5-1.0)	344	34 (7.5)	0.7 (0.37-1.2)	0.1714			
High-average (1.0-2.0)	292	30 (7.6)	0.7 (0.4-1.3)	0.2091			
High (>2.0)	291	35 (10.9)	1				
Marital status							
Married/cohabiting	777	80 (8.7)	1				
Previously married/Never married	494	37 (6.7)	0.8 (0.5-1.2)	0.2566			
Employment status				0.0058			
Employed/student	729	58 (5.9)	1				
Homemaker/retired	331	42 (11.8)	2.2 (1.4-3.5)	0.0014			
Unemployed	211	17 (10.1)	1,8 (0.8-4.4)	0.1839			
Anxiety disorder							
No	435	28 (7.3)	1		1		
Yes	836	89 (8.1)	1.1 (0.7-1.7)	0.585	3.5 (1.6-7.8)		0.0019
Mood disorder							
No	704	35 (3.2)	1		1		
Yes	567	82 (14.7)	5.2 (2.6-10.3)	<0.0001	5.7 (2.5-13.0)		<0.0001
SUD^a^							
No	1108	104 (7.8)	1		1		
Yes	163	13 (7.9)	1.0 (0.5-2.1)	0.984	2.9 (1.5-5.7)	0.0019	
ICD^b^							
No	1080	101 (8.2)	1				
Yes	191	16 (5.8)	0.7 (0.4-1.3)	0.2634			
Comorbidity							
No	731	48 (5.6)	1		1		
Yes	540	69 (11.2)	2.1 (1.3-3.5)	0.0035	0.4 (0.2-1.0)	0.0255	
Severity							
Mild	397	19 (3.7)	1				
Serious/Moderate	874	98 (10.0)	2.8 (1.7-4.8)	0.0001			
Service use							
No	935	15 (1.3)	1		1		
Yes	336	102 (29.8)	31.2 (19,3-50.4)	<0.0001	25.0 (13.7-45.6)	<0.0001	
Health insurance							
No	798	56 (6.1)	1				
Yes	473	61 (10.7)	1.9 (1.0-3.4)	0.0505			

Weighted proportions. OR, odds ratio; CI, confidence interval. ^a^Substance use disorders. ^b^Impulse control disorders. Model 1: crude. Model 2: All variables were analyzed together.

**Table 5:** Twelve-month prevalence of benzodiazepine use according to the DSM-IV/WMH-CIDI diagnosis by sex. Results from the São Paulo Megacity, São Paulo, Brazil (N=1,271).

		At least one BZD					Exclusive use		
Mental health disorder	Total	N (%)	SE	OR (95% CI)	*X^2^*	*p*-value	N (%)	SE	OR (95% CI)
Anxiety Disorders									
Panic disorder	61	17 (33.7)	8.9	1.7 (0.3-10.4)	0.4	0.542	04 (9.8)	4.2	0.4 (0.0-3.8)
Generalized anxiety disorder	128	08 (8.4)	3.6	0.5 (0.1-3.3)	0.6	0.4391	03 (4.3)	3.1	0.1 (0.0-1.9)
Specific phobia	471	54 (7.7)	1.3	0.8 (0.3-2.3)	0.2	0.6757	19 (2.8)	0.7	0.8 (0.2-3.4)
Social phobia	174	23 (10.4)	3.1	2.3 (0.6-9.8)	1.3	0.25	05 (2.1)	1.1	0.8 (0.1-5.2)
Agoraphobia without panic	88	16 (13.5)	3.3	9.9 (0.9-104.6)	3.6	0.0573	04 (3.9)	2.2	2.3 (0.2-26.5)
Post-traumatic stress disorder	80	10 (6.6)	2.5	0.7 (0.1-4.7)	0.18	0.6714	03 (2.5)	1.6	0.1 (0-0.8)
Obsessive-compulsive disorder	155	19 (8.8)	2.5	7.0 (1.6-30.0)	6.8	0.0094	04 (1.4)	0.8	0.4 (0.0-3.2)
Adult separation anxiety	95	12 (12.6)	4.3	-	-	-	04 (4.2)	2.4	-
Any anxiety disorder	836	89 (8.1)	1.0	1.1 (0.5-2,52)	0.0	0.8466	31 (2.9)	0.6	0.6 (0.2-1.5)
Mood Disorders									
Major depressive disorder	488	62 (13.4)	2.2	1.4 (0.4-4.28)	0.3	0.5879	17 (4.0)	1.0	0.9 (0.2-4.0)
Dysthymia	62	09 (11.8)	5.6	7.5 (0.5-104.2)	2.2	0.1349	02 (2.7)	1.9	-
Bipolar I and II disorders	73	19 (23.3)	5.5	1.4 (0.5-4.1)	0.4	0.5106	04 (4.5)	2.8	2.3 (0.5-10.7)
Any mood disorder	567	82 (14.7)	2.3	1.3 (0.5-3.6)	0.3	0.6112	21 (4.0)	1.0	1.1 (0.3-4.1)
Substance Use Disorders									
Alcohol abuse	134	10 (7.8)	3.1	2.0 (0.5-8.05)	0.9	0.3492	04 (2.8)	1.7	0.9 (0.1-11.9)
Alcohol dependence	64	07 (13.2)	6.3	2.1 (0.4-9.5)	0.8	0.3614	02 (3.9)	3.1	-
Drug abuse	31	03 (6.6)	3.6	8.2 (1.9-36.4)	7.8	0.0054	01 (2.4)	2.2	-
Drug dependence	21	03 (11.7)	7.4	9.3 (1.5-58.8)	5.6	0.0176	01 (2.6)	2.6	-
Any substance use disorder	163	13 (7.9)	2.5	3.6 (0.9-15.1)	3.1	0.0763	04 (2.2)	1.3	0.8 (0.1-10.3)
Impulse-control Disorders									
Attention deficit disorder	45	07 (13.3)	6.0	17.5 (2.1-146.8)	6.98	0.0083	03 (10.1)	5.8	11.3 (1.4-90.6)
Oppositional-defiant disorder	20	01 (3.8)	3.8	-	-	-			
Conduct disorder	17	03 (15.9)	9.3	-	-	-	01 (8.4)	8.0	-
Intermittent explosive disorder	137	10 (4.6)	1.5	4.1 (0.7-23.3)	2.6	0.1096	03 (1.2)	0.7	2.3 (0.2-30.9)
Any impulse-control disorder	191	16 (5.8)	1,7	5.6 (1.1-27.7)	4.4	0.0352	06 (2.9)	1.1	4.2 (0.5-37.6)
Any 12-month Disorder									
Any	1271	117 (7.8)	0.8	1.5 (0.7-3.2)	0.9	0.3563	40 (2.7)	0.4	0.8 (0.4-1.8)
0 Disorders	1664	45 (1.9)	0.6	13.0 (4.1-41.3)	19.0	<0.0001	25 (1.4)	0.6	20.9 (5.4-81.9)
1 Disorder	731	48 (5.6)	0.9	1.2 (0.5-3.0)	0.1	0.7294	20 (2.2)	0.5	0.6 (0.2-1.8)
2 Disorders	262	19 (6.1)	1.0	1.2 (0.2-6. 3)	0.1	0.8004	08 (2.6)	1.0	1.4 (0.2-9.8)
3+ Disorders	278	50 (16.3)	3.0	2.3 (0.9-6.1)	3.0	0.0836	12 (4.6)	1.4	1.1 (0.2-5.2)
Severity									
Serious	465	77 (15.3)	2.1	1.3 (0.6-2.9)	0.4	0.5226	-	-	-
Moderate	409	21 (4.2)	1.1	2.2 (0.4-10.9)	0.9	0.3438	-	-	-
Mild	397	19 (3.7)	0.8	3.4 (0.8-14.4)	2.7	0.0999	-	-	-
None	1664	45 (1.9)	0.6	13.0 (4.1-41.3)	19.0	<0.0001	-	-	-

Weighted proportions. OR, odds ratio; CI, confidence interval. Sex comparison: males were used as the reference group.
